# Metabolic Dysfunction and Inflammation in Diabetic Retinopathy: Insights From Metabolomics and Cytokine Analysis

**DOI:** 10.1155/mi/8850352

**Published:** 2026-08-04

**Authors:** Diyang Ke, Yu Wu, Daizai Lei, Xiaozhan Zheng, Ningning Tang, Lingjuan Liu, Li Jiang, Zhijie Niu, Shaoyang Zhang, Wenjing He, Chaolan Shen, Yuanjun Qin, Haibin Zhong, Ling Cui, Fan Xu, Fen Tang

**Affiliations:** ^1^ Department of Ophthalmology, The People’s Hospital of Guangxi Zhuang Autonomous Region and Guangxi Key Laboratory of Eye Health and Guangxi Health Commission Key Laboratory of Ophthalmology and Related Systemic Diseases Artificial Intelligence Screening Technology & Institute of Ophthalmic Diseases, Guangxi Academy of Medical Sciences, Nanning, Guangxi, China; ^2^ Guilin Medical University, Guilin, Guangxi, China, glmc.edu.cn; ^3^ Department of Otolaryngology-Head and Neck Surgery, The First Affiliated Hospital of Guangxi Medical University, Nanning, Guangxi, China, gxmu.edu.cn

**Keywords:** diabetic retinopathy, metabolism pathway, metabolomics analysis, ocular inflammation, VEGF

## Abstract

**Purpose:**

Diabetic retinopathy (DR) is a leading vision‐threatening disease and a common complication of diabetes. However, the understanding of how metabolic dysfunction correlates with ocular inflammation in DR remains poorly understood. Therefore, in this study, we investigated the association between these two factors in DR.

**Methods:**

Using untargeted metabolomics analysis and mass spectrometry detection, the plasma metabolic characteristics of 27 DR patients (15 with nonproliferative diabetic retinopathy [NPDR] and 12 with proliferative diabetic retinopathy [PDR]) and 8 healthy controls (HCs) were examined. Additionally, the concentrations of inflammatory factors and vascular endothelial growth factor (VEGF) in their aqueous humor were measured. Further analysis was conducted to evaluate the correlation between metabolic disorders and abnormalities in inflammatory factors and VEGF in the aqueous humor.

**Results:**

Metabolomic analysis revealed significant metabolic dysregulation in DR, with PDR exhibiting particularly pronounced alterations compared to NPDR and HC. ROC analysis identified creatinine, glutamine, indoleacetic acid, and methionine as reliable biomarkers for distinguishing PDR. Elevated cytokines, such as IL‐6, IL‐8, and VEGF, were significantly correlated with specific metabolism pathways.

**Conclusion:**

Our study highlights potential biomarkers for DR stratification and provide novel insights into the link between metabolic dysfunction and ocular inflammation/ischemia in PDR.

## 1. Introduction

Diabetes mellitus (DM) is a global health challenge, with its prevalence more than doubling over the past three decades, making it the ninth leading cause of death worldwide [1]. Among the various complications associated with diabetes, DR stands out as one of the most common and severe microvascular complications, leading to blindness in the working‐age population [2]. DR progresses from NPDR to the more severe PDR, which can result in gradual loss of both peripheral and central vision [3]. Given the projected rise in DR prevalence—from 103 million in 2020 to 161 million by 2045—identifying risk factors and biomarkers is crucial for early diagnosis and intervention [4].

Emerging evidence suggests that inflammation plays a central role in DR development and progression. Elevated inflammatory cytokines in the vitreous and aqueous humor, such as interleukin‐1β (IL‐1β), interleukin‐6 (IL‐6), interleukin‐8 (IL‐8), and tumor necrosis factor‐α (TNF‐α), are vital biomarkers for ocular inflammation and are also recognized as the key drivers in the development of DR [5].

Metabolomics are pivotal tool, offering insights into biomarkers and mechanisms in DR [6]. Previous studies have suggested that metabolic dysfunction, particularly hyperglycemia, is a key regulator of inflammation in DR through pathways like glycolytic overload, oxidative stress, and ER‐mitochondrial dysfunction [7, 8]. Recent advances in immunometabolism have further illuminated the complex interplay between host metabolism and immune responses [9, 10]. Despite these advances, our understanding of the correlation between metabolic changes and immune responses in DR patients remains limited.

In this study, we used ultra‐high performance liquid chromatography‐tandem mass spectrometry (UPLC‐MS/MS) to characterize the plasma metabolic profiles of healthy controls (HCs), patients with NPDR and PDR. In the present study, we aim to explore the global metabolic landscape and changes in DR patients, especially between NPDR and PDR, and to identify reliable biomarkers for predicting DR disease severity. In parallel, we quantified several key cytokines in the aqueous humor of these patients. By integrating cytokine measurements with metabolomic analysis, this investigation sought to elucidate the relationship between metabolic dysregulation and cytokine upregulation, potentially uncovering regulatory interactions between altered metabolic pathways and inflammatory mediators in DR pathogenesis.

## 2. Methods

### 2.1. Patient Characteristics

Plasma samples were collected from 27 patients diagnosed with DR, including 15 with NPDR and 12 with PDR, along with 8 HCs. Additionally, aqueous humor was collected for cytokine analysis. All samples were collected in a fasting state on the same day prior to surgery. Controls were age‐matched and recruited from the outpatient clinic during preoperative screening for cataract surgery. The demographic characteristics of the study participants are summarized in Table 1. Patients aged 18 and above were recruited between January 2020 and March 2023 from the ophthalmology outpatient clinic at Guangxi Zhuang Autonomous Region People’s Hospital. Diagnoses of NPDR and PDR were confirmed by ophthalmologists. All patients had not received anti‐VEGF therapy or laser therapy. Exclusion criteria included systemic inflammatory diseases (e.g., infectious, autoimmune, rheumatic, severe atherosclerosis, and other inflammatory skin diseases) and the use of systemic immunosuppressive or immunomodulatory therapies. The study adhered to the principles of the Declaration of Helsinki, and ethics approval was obtained from the People’s Hospital of Guangxi Zhuang Autonomous Region (Number: KY‐GZR‐2023‐106). All participants provided informed consent for the use of their data in the research.

**Table 1:** Clinical and demographic characteristics of patients.

Characteristic	HC (*n* = 8)	NPDR (*n* = 15)	PDR (*n* = 12)	P1	P2	P3
Age (years)	55.875 (41–68)	55.467 (39–71)	60.58 (49–74)	NS	NS	NS
Gender (male)	3	7	5	—	—	—
Diabetes duration	0	3–20 (10.7)	5–20 (13.3)	—	—	—
BMI (kg/m^2^)	25.1 (22.5–27.2)	23.8 (19.1–27.7)	24.0 (19.0–27.9)	NS	NS	NS
HDL‐c (mmol/L)	0.93 (0.67–1.07)	1.27 (0.80–1.84)	0.88 (0.55–1.25)	0.019	NS	NS
LDL‐c (mmol/L)	1.14 (0.31–2.44)	5.26 (1.34–7.65)	4.67 (1.49–7.58)	0.001	0.001	NS
TC (mmol/L)	3.25 (1.9–4.8)	7.81 (4.5–11.4)	6.22 (2.6–11.7)	0.001	0.025	0.05
FPG (mmol/L)	5.22 (4.3–6.1)	5.67 (3.6–9)	5.99 (3.8–9)	NS	NS	NS
UACR (mg/g)	4.6 (3–27)	82.7 (37–120)	82.0 (15–143)	0.001	0.001	NS
HbA1c (%)	4.60 (4.1–5.5)	6.51 (3.8–9.2)	6.95 (2.1–9.5)	0.011	0.004	NS

*Note:* P1: the *p*‐value between NPDR vs HC; P2: the *p*‐value between PDR vs. HC; P3: the *p*‐value between PDR vs. NPDR.

Abbreviations: BMI, body mass index; FPG, fasting plasma glucose; HbA1c, glycated hemoglobin; HDL‐c, high‐density lipoprotein cholesterol; LDL‐c, low‐density lipoprotein cholesterol; NS, not significant; TC, total cholesterol; UACR, urine albumin‐to‐creatinine ratio.

### 2.2. Plasma and Aqueous Humor Collection

Whole blood samples were collected using EDTA anticoagulant tubes and gently inverted 8–10 times to ensure proper mixing. The samples were then centrifuged at 1600 × *g* for 10 min at 4°C. The resulting plasma supernatant was carefully transferred into cryovials and stored at −80°C for long‐term preservation.

Aqueous humor samples were obtained under strict aseptic conditions during cataract surgery or intraocular injection surgery. After disinfection and sterile draping, an insulin syringe (30‐gauge needle) was used to gently withdraw about 100–200 μL of fluid in the anterior chamber. The aqueous humor samples were immediately frozen at −80°C until further analysis for cytokines. Whole blood and aqueous humor were collected on the same day.

### 2.3. Untargeted Metabolomic

Sample preparation was conducted at the BGI Genomics metabolomics facility. Plasma samples were thawed slowly at 4°C, and 100 μL of each sample was placed in a 96‐well plate. An extraction solution (methanol:acetonitrile = 2:1, v:v, precooled to −20°C) containing 10 μL of internal standard was added to each well. The samples were vortexed for 1 min, left at −20°C for 2 h, and then centrifuged at 4°C, 4000 rcf for 20 min. After centrifugation, 300 μL of the supernatant was collected and dried using a freeze vacuum concentrator. The dried samples were reconstituted in 150 μL of reconstitution solution (methanol:water = 1:1, v:v), vortexed for 1 min, and centrifuged at 4°C, 4000 rcf for 30 min. The supernatant was transferred to sampling vials for analysis. A pooled quality control (QC) sample was prepared by mixing 10 μL of each sample’s supernatant to assess the repeatability and stability of the liquid chromatography‐mass spectrometry (LC‐MS) analysis. Metabolite identification was based on m/z accuracy and MS/MS fragmentation patterns, with all metabolites annotated at MSI level 2. The mass error for all metabolites was less than 5 ppm. Data analysis was performed using Thermo Scientific Compound Discoverer and Thermo Scientific TraceFinder software.

The raw mass spectrometry data acquired from LC‐MS/MS were imported into Compound Discoverer 3.1 (Thermo Fisher Scientific, USA) for data processing. This process mainly included peak extraction, intra‐ and inter‐group retention time correction, adduct ion merging, missing value imputation, background peak labeling, and metabolite identification. Lastly, information such as compound molecular weight, retention time, peak area, and identification results were exported. Metabolite identification combined multiple databases, including the BGI Metabolomics Database, mzCloud, and ChemSpider. The main parameters for metabolite identification were mass deviation of the parent ion < 5 ppm, mass deviation of the fragment ion <10 ppm, and retention time deviation <0.2 min.

The results exported from Compound Discoverer 3.1 were imported into metaX for data preprocessing, which mainly included: (1) normalizing the data using the probabilistic quotient normalization method to obtain relative peak areas; (2) applying QC‐RLSC local polynomial regression fitting to correct signal batch effects; (3) removing compounds with a coefficient of variation (CV) of relative peak areas greater than 30% in all QC samples.

### 2.4. Cytokines Measurements

The concentrations of these cytokines—IL‐1β, IL‐6, IL‐8, IL‐12p70, TNF‐α, IL‐10, and VEGF—were measured in Beijing Giantmed Medical Diagnostics Laboratory using a cytometric bead array (CBA). Immunoassays were conducted according to the Immune Monitoring Core’s Standard Operating Procedures (SOP), with data fitting performed using a 5‐parameter log curve in Bioplex Manager 6.0. Standard curves and QCs provided by the supplier ensured the accuracy and reliability of cytokine quantification.

### 2.5. Statistical Analysis

Protein concentrations were normalized for each sample prior to analysis. MetaboAnalyst 6.0 (Quebec, Canada) was used for noise filtering, data transformation, and scaling of peak intensities. Statistical analysis of normalized intensities was conducted using the Student’s *t*‐test for continuous variables. Fold change (FC) intensity was calculated by dividing the normalized intensity of each sample by the average normalized intensity of the HC group. False discovery rate (FDR) correction was applied to adjust for multiple comparisons in metabolite analysis. Multivariate analyses, including principal component analysis (PCA), partial least squares discriminant analysis (PLS‐DA), orthogonal partial least squares discriminant analysis (OPLS‐DA), and K‐means clustering, were used to identify distinct patient clusters. Receiver operating characteristic (ROC) curves were generated in MetaboAnalyst 6.0 to evaluate potential biomarkers. Metabolite enrichment analysis was conducted using the Human Small Molecule Pathway Database (SMPDB). All statistical analyses were performed using R or MetaboAnalyst 6.0. Continuous and categorical variables were compared using two‐tailed Student’s *t*‐tests and Pearson *χ* ^2^ tests, respectively. The Pearson rank correlation coefficient was calculated to assess the relationships between metabolite levels and cytokines. To construct robust and interpretable association networks between metabolites and cytokines, we employed the Debiased Sparse Partial Correlation (DSPC) method. GraphPad Prism (8.0.1) and SPSS (25.0) were applied for data analysis. A *p*‐value of less than 0.05 was regarded as statistically significant.

## 3. Results

### 3.1. Global Metabolic Features in NPDR and PDR Patients

**Figure 1:**
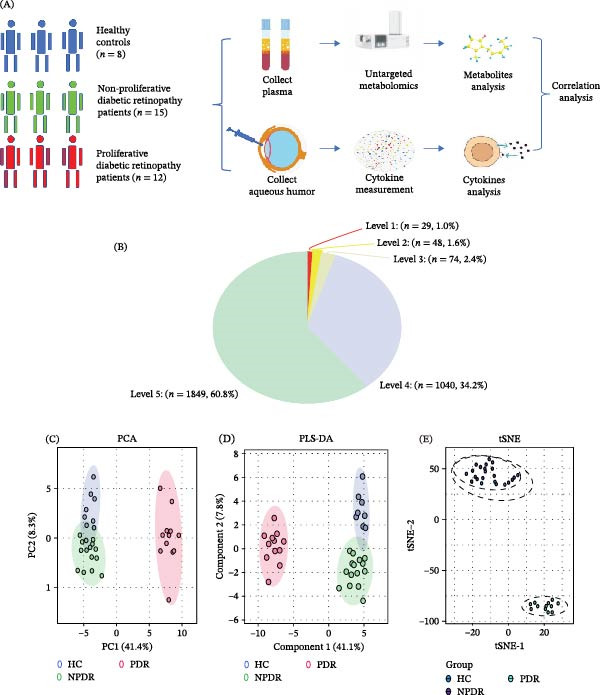
Study workflow and global metabolic profiling in NPDR and PDR patients. (A) Schematic overview of the study design, including subject enrollment, sample collection, and metabolomics analysis. (B) Metabolite credibility grading pie chart, classifying detected metabolites into five levels based on credibility. (C) Principal component analysis (PCA) score plot showing the distribution of NPDR, PDR, and HC samples based on global metabolic profiles. (D) Partial least squares discriminant analysis (PLS‐DA) score plot, demonstrating clear separation between groups. The model was established using three principal components, with cumulative *R* ^2^ = 0.964, *Q* ^2^ = 0.801, and accuracy = 90.9%. (E) t‐SNE clustering visualization of three groups.

We enrolled a total of 35 subjects, comprising 15 patients with NPDR, 12 patients with PDR, and 8 HCs. Plasma samples were collected for untargeted metabolomics analysis, and aqueous humor samples were collected for cytokine analysis. The study workflow is depicted in Figure 1A. Through the LC‐MS/MS system, we identified 1841 metabolites in positive ion mode and 790 metabolites in negative ion mode in the plasma, and 77 metabolites with the highest identification confidence (levels 1 and 2) were selected for further analysis (Figure 1B).

To investigate the metabolic differences, we conducted PCA, which revealed a clear separation between PDR patients and the other groups, with partial overlap between the control and NPDR groups (Figure 1C). We then used PLS‐DA and t‐SNE clustering to further characterize differential metabolites. The PLS‐DA permutation test (2000 iterations) yielded *R* ^2^ and *Q* ^2^ values of 0. and 0.87, respectively, indicating strong predictive ability with no overfitting (*p*  < 0.05) (Figure 1D). The t‐SNE plot demonstrated a distinct separation of PDR samples from the other groups, while NPDR and control group profiles showed some similarity with partial overlap. These results collectively indicate that compared to HCs, both NPDR and PDR groups exhibit plasma metabolic disturbances, with PDR demonstrating particularly pronounced metabolic alterations, whereas NPDR showed more subtle changes relative to controls.

**Figure 2:**
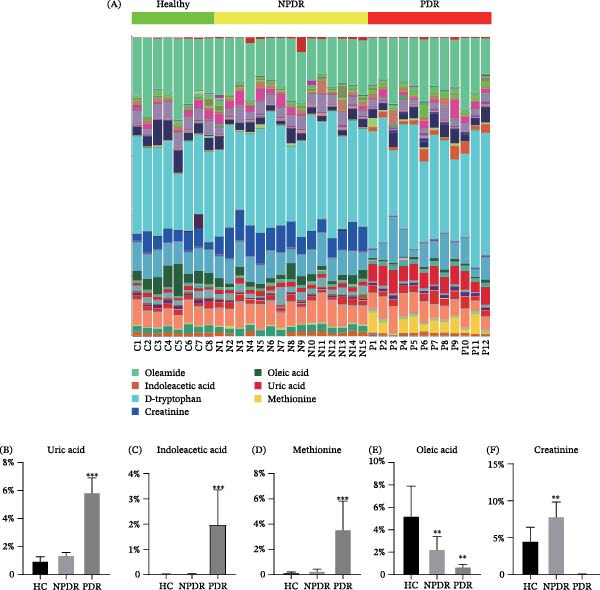
Differential metabolite distribution among HC, NPDR, and PDR groups. (A) Stacked bar chart illustrating the proportion of major metabolite classes in NPDR, PDR, and HC groups. (B–F) Bar charts showing the relative abundance of selected metabolites in each group. Statistical significance was determined by Student’s *t*‐test.  ^∗^ *p* < 0.05,  ^∗∗^ *p* < 0.01,  ^∗∗∗^ *p* < 0.001. (B) Uric acid percentage. (C) Indoleacetic acid percentage. (D) Methionine percentage. (E) Oleic acid percentage. (F) Creatinine percentage.

Among the 77 metabolites, we observed significant differences in distribution between the HC, NPDR, and PDR groups. The pie chart in Figure 2A illustrates the metabolic alterations among these three groups. D‐Tryptophan represented the highest proportion in all three groups, accounting for 28.3% (HC), 33.1% (NPDR group), and 35.2% (PDR group) of the total metabolite composition, respectively. Additionally, oleamide also maintained relatively high proportions across all three study groups. Notably, we identified several metabolites that were predominantly present in the PDR group while being absent or minimally detected in the HC or NPDR groups, including uric acid (Figure 2B), indoleacetic acid (Figure 2C), and methionine (Figure 2D). Conversely, oleic acid (Figure 2E) and creatinine (Figure 2F) showed the lowest proportions in the PDR group. Interestingly, creatinine levels were highest in the NPDR group while decreasing in the PDR group.

Overall, these findings suggest that while NPDR shows minor metabolic disturbances, its profile still resembles that of HC, whereas PDR patients exhibit significant metabolic dysregulation compared to both NPDR and controls.

### 3.2. Significant Regulated Metabolites in NPDR and PDR Patients

The global metabolic patterns across the three groups are illustrated in the heatmap in Figure 3A. We can observe that the global metabolic patterns of HC and NPDR groups appear relatively similar, while the PDR pattern differs substantially. Notably, 8 metabolites showed consistently elevated expression in all PDR subjects while displaying low expression in all HC and NPDR subjects. These include D‐ornithine, pipecolic acid, threonic acid, L‐tyrosine, L‐tryptophan, indoleacetic acid, uric acid, and methionine (Figure 3A, red square). Conversely, 11 metabolites exhibited consistently low expression in all PDR subjects while showing high expression in all HC and NPDR subjects, including pilocarpine, hypoxanthine, and histidine, and so on (Figure 3A, blue square).

Figure 3Differentially expressed metabolites in NPDR and PDR patients. (A) Heatmap illustrating global metabolic patterns across HC, NPDR, and PDR groups using Euclidian distance measure and Ward clustering algorithm. Metabolites with consistently high expression in PDR compared to NPDR and HC are highlighted with red box, while those with consistently low expression in PDR are marked with blue box. (B) Volcano plots showing differentially expressed metabolites (DEMs) between NPDR vs. HC comparison. (C) Volcano plots showing DEMs between PDR vs. HC. (D) Volcano plots showing DEMs between PDR vs. NPDR. The *X*‐axis represents log2 fold change (FC), and the *Y*‐axis represents ‐log10(*p*‐value). The top 5 statistically significant upregulated metabolites and downregulated metabolites (log2FC > 1, *p* < 0.01) are labeled. Gray dots indicate nonsignificant metabolites.

**Figure:**
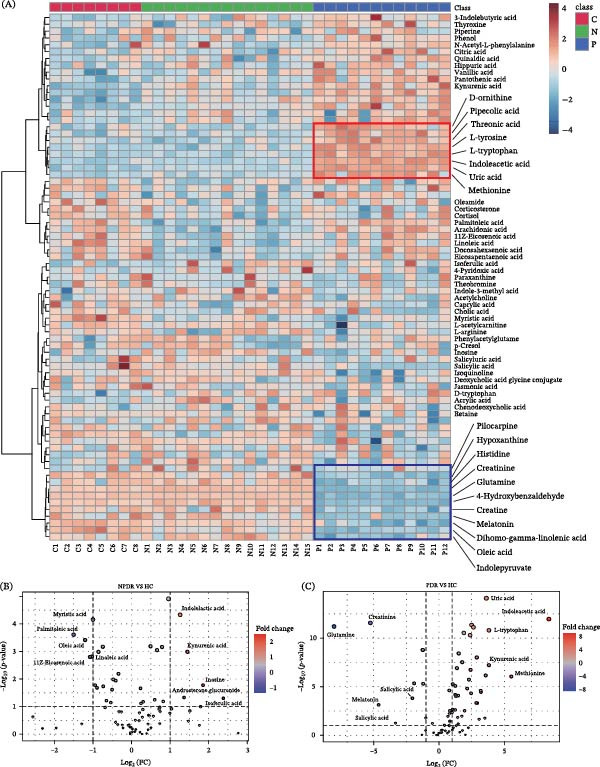

**Figure:**
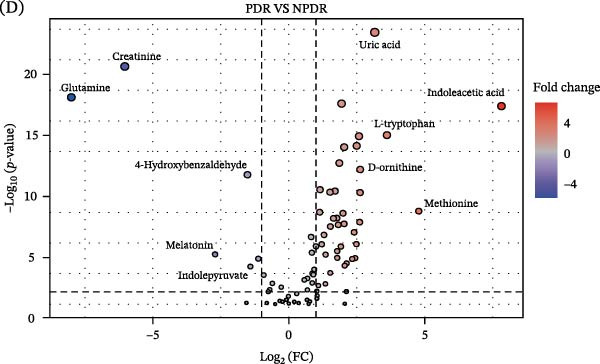

Further analysis between two group comparisons was performed to identify statistically significant differential expressed metabolites (DEMs). Selection criteria were based on variable importance in the projection (VIP), FC from univariate analysis, and *t*‐test results. We defined DEMs as those meeting three criteria: (1) VIP ≥ 1; (2) Log2FC ≥ 1 or ≤ −1; and (3) *p*‐value < 0.01.

The volcano plot in Figure 3B illustrates the differential metabolites between NPDR and HC groups, revealing 6 upregulated DEMs and 5 downregulated DEMs that met statistical significance. Figure 3C displays the differential metabolites between PDR and HC groups, showing a substantially higher number of DEMs compared to the previous comparison, with 37 upregulated DEMs and 9 downregulated DEMs. Additionally, we analyzed the DEMs between PDR and NPDR groups (Figure 3D). Similar to the PDR versus HC comparison, upregulated DEMs far outnumbered downregulated DEMs, with the most significantly altered DEMs largely overlapping with those identified in the PDR versus HC comparison. Overall, we have compiled and summarized the top 5 upregulated and downregulated DEMs from each pairwise comparison in Table 2. The complete list of DEMs has been included in Supporting Information 1: File 1–Supporting Information 3: File 3.

**Table 2:** The top 5 upregulated and downregulated DEMs among HC, NPDR, and PDR.

Comparison	Metabolite	log_2_(FC)	*p*‐Value	Regulation
NPDR vs. HC	Upregulated	—	—	—
	Isoferulic acid	2.381	0.049931	Up
	Inosine	1.8538	0.016818	Up
	Kynurenic acid	1.4481	0.0010295	Up
	Androsterone glucuronide	1.3629	0.046829	Up
	Indolelactic acid	1.2603	0.0000466	Up
	Downregulated	—	—	—
	Palmitoleic acid	−1.5027	0.00024598	Down
	Oleic acid	−1.2059	0.00038426	Down
	11 Z‐eicosenoic acid	−1.08	0.0015863	Down
	Linoleic acid	−1.0267	0.0015299	Down
	Myristic acid	−1.0015	0.0000691	Down
PDR vs. HC	Upregulated	—	—	—
	Indoleacetic acid	8.4150	1.07E−12	Up
	Methionine	5.5312	8.93E−07	Up
	L‐tryptophan	3.8193	1.56E−11	Up
	Kynurenic acid	3.8141	6.07E−08	Up
	Uric acid	3.6220	7.61E−15	Up
	Downregulated	—	—	—
	Glutamine	−8.0423	6.25E−12	Down
	Creatinine	−5.2615	2.71E−12	Down
	Melatonin	−4.6278	0.0007	Down
	Dihomo‐gamma‐linolenic acid	−2.0400	0.0002	Down
	Oleic acid	−1.9091	4.5229E−6	Down
PDR vs. NPDR	Upregulated	—	—	—
	Indoleacetic acid	7.8198	6.21E−17	Up
	Methionine	4.7789	2.43E−08	Up
	L‐tryptophan	3.6080	1.46E−14	Up
	Uric acid	3.1608	5.64E−23	Up
	D‐ornithine	2.6232	9.53E−12	Up
	Downregulated	—	—	—
	Glutamine	−7.9985	1.17E−17	Down
	Creatinine	−6.0331	3.51E−20	Down
	Melatonin	−2.7126	8.46E−05	Down
	4‐Hydroxybenzaldehyde	−1.5217	2.57E−11	Down
	Indolepyruvate	−1.4137	0.0008	Down

Abbreviation: DEMs, differentially expressed metabolites.

### 3.3. Plasma Biomarkers and Enriched Metabolic Processes in NPDR and PDR

In order to identify reliable plasma biomarkers for predicting NPDR and PDR, we conducted ROC curve analysis on the DEMs. In NPDR patients, oleic acid (AUC = 0.958), myristic acid (AUC = 0.95), palmitoleic acid (AUC = 0.925), kynurenic acid (AUC = 0.908), and indoleacetic acid (IAA) (AUC = 0.717) were identified as reliable biomarkers with high sensitivity and specificity (Figure 4A).

Figure 4Plasma biomarkers and enriched metabolic pathways in NPDR and PDR patients. (A) Receiver operating characteristic (ROC) curves identified reliable plasma biomarkers distinguishing NPDR patients from HC, including oleic acid, myristic acid, palmitoleic acid, kynurenic acid, and indoleacetic acid (IAA). (B) Pathway enrichment analysis of differentially expressed metabolites (DEMs) in NPDR. Upregulated DEMs are primarily enriched in androstenedione metabolism. (C) Downregulated DEMs are predominantly associated with alpha linolenic acid and linoleic acid metabolism in NPDR. (D) ROC curves for the top four identified plasma biomarkers distinguishing PDR patients from HC, including creatinine, glutamine, indoleacetic acid, and methionine. (E) Pathway enrichment analysis of DEMs in PDR. Upregulated DEMs are significantly enriched in tryptophan metabolism, and so on. (F) Downregulated DEMs are primarily associated with purine metabolism and phenylacetate metabolism in PDR.

**Figure:**
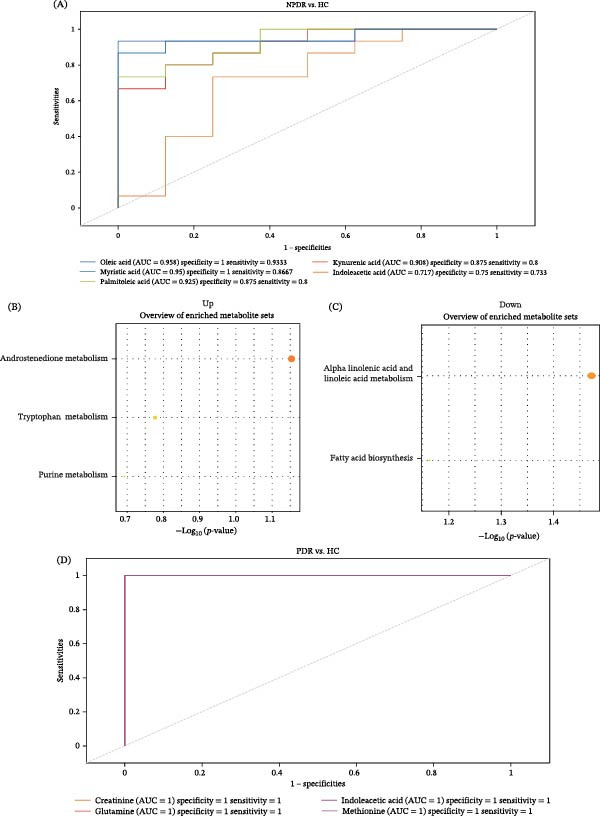

**Figure:**
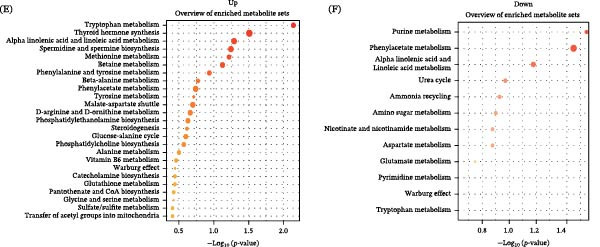

We performed pathway enrichment analysis of the metabolite sets using MetaboAnalyst 6.0 and the SMPDB, analyzing upregulated and downregulated DEMs separately. Results revealed that upregulated DEMs in NPDR were primarily enriched in androstenedione metabolism (Figure 4B), while downregulated DEMs were predominantly associated with alpha linolenic acid and linoleic acid metabolism (Figure 4C).

For PDR patients, identified reliable biomarkers included creatinine (AUC = 1), glutamine (AUC = 1), indoleacetic acid (AUC = 1), and methionine (AUC = 1) (Figure 4D). Similarly, we conducted GO enrichment analysis on both upregulated and downregulated DEMs. The upregulated DEMs in PDR were significantly enriched in tryptophan metabolism, thyroid hormone synthesis, alpha linolenic acid and linoleic acid metabolism, and spermidine and spermine biosynthesis, and so on (Figure 4E). In contrast, downregulated DEMs were primarily enriched in purine metabolism and phenylacetate metabolism (Figure 4F).

### 3.4. Correlation Between Regulated Metabolomics and Cytokines

**Figure 5:**
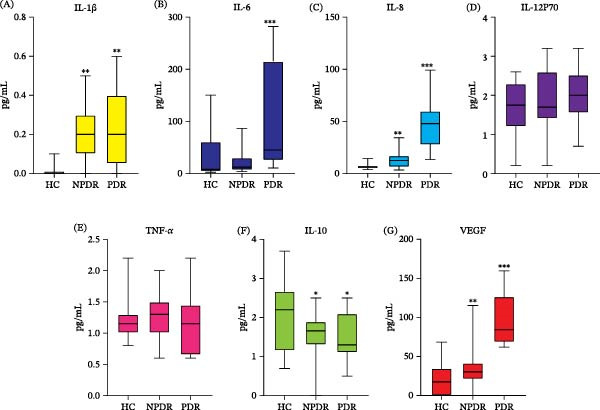
Intraocular cytokine and VEGF concentrations in NPDR and PDR patients. (A–G) Bar charts of cytokines and VEGF levels in the aqueous humor of HC, NPDR, and PDR groups. (A) IL‐1β, (B) IL‐6, (C) IL‐8, (D) IL‐12P70, (E) TNF‐α, (F) IL‐10, and (G) VEGF. Statistical significance was determined using Student’s *t*‐test.  ^∗^ *p* < 0.05,  ^∗∗^ *p* < 0.01,  ^∗∗∗^ *p* < 0.001.

To further explore the correlation between plasma metabolism dysfunction and ocular inflammation status in DR, we measured the concentrations of several key cytokines and angiogenic factor VEGF in the aqueous humor across the three groups. As shown in Figure 5, several proinflammatory cytokines were greatly elevated in DR, particularly in the PDR group. IL‐1β (Figure 5A) exhibited moderate elevation in both NPDR and PDR groups, whereas IL‐6 (Figure 5B) and IL‐8 (Figure 5C) showed dramatic increases in PDR but only mild elevation in NPDR. Additionally, other factors such as IL‐12P70 (Figure 5D), TNF‐α (Figure 5E), and IL‐17 (data not shown) did not demonstrate significant increases. Conversely and expectedly, the anti‐inflammatory cytokine IL‐10 showed decreased expression in DR, especially in PDR. As anticipated, the most important intraocular angiogenic factor VEGF was also significantly upregulated in DR, particularly in PDR, with mean concentrations increasing more than 4‐fold (Figure 5G). These intraocular inflammatory factors and VEGF levels reflect the severity of intraocular inflammation and ischemia in individual DR patients.

Independent correlation analyses using Pearson’s method were conducted separately for NPDR (Figure 6A) and PDR (Figure 6B) patients, with correlations considered significant at FDR < 0.05. These analyses revealed associations between each inflammatory cytokine/VEGF and multiple metabolites in both disease stages (Figure 6A,B). To elucidate the predominant metabolic pathways associated with altered inflammatory mediators, we performed pathway enrichment analysis on the significantly correlated metabolites in PDR. The results, visualized as bubble charts (Figure 6C–G), demonstrated distinct pathway associations: the pronounced IL‐6 elevation correlated primarily with spermidine and spermine biosynthesis and betaine metabolism pathways (Figure 6C); the marked IL‐8 increase associated significantly with pantothenate and CoA biosynthesis and beta‐alanine metabolism (Figure 6D); the substantial IL‐10 reduction correlated with dysregulation in steroidogenesis and phosphatidylcholine biosynthesis pathways; the moderate IL‐1β upregulation showed significant associations with phenylacetate metabolism and sulfate/sulfite metabolism; and notably, the substantial VEGF elevation correlated strongly with alterations in spermidine and spermine biosynthesis and vitamin B6 metabolism pathways (Figure 6G). These findings establish clear links between specific metabolic pathway perturbations and the inflammatory/angiogenic profile characteristic of DR progression.

Figure 6Correlation analysis between cytokines and metabolites. (A,B) Correlation analysis between metabolites and cytokine levels in NPDR (A) and PDR (B) patients. The color gradient represents the Pearson correlation coefficient, with red indicating positive correlation and blue indicating negative correlation. Point size reflects statistical significance, with larger points denoting higher confidence (FDR < 0.05). (C–G) Pathway enrichment analysis of metabolites significantly correlated with inflammatory cytokines and VEGF in PDR patients. (C) IL‐6 elevation‐associated pathways. (D) IL‐8 elevation‐associated pathways. (E) IL‐10 downregulation‐associated pathways. (F) IL‐1β elevation‐associated pathways. (G) VEGF upregulation‐associated pathways.

**Figure:**
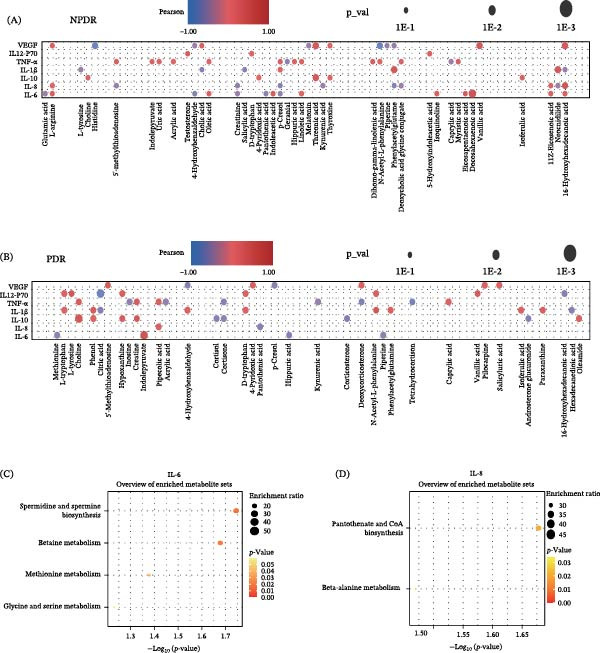

**Figure:**
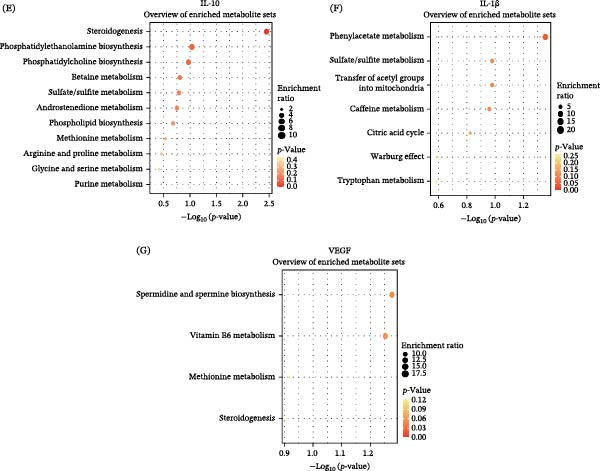

**Figure 7:**
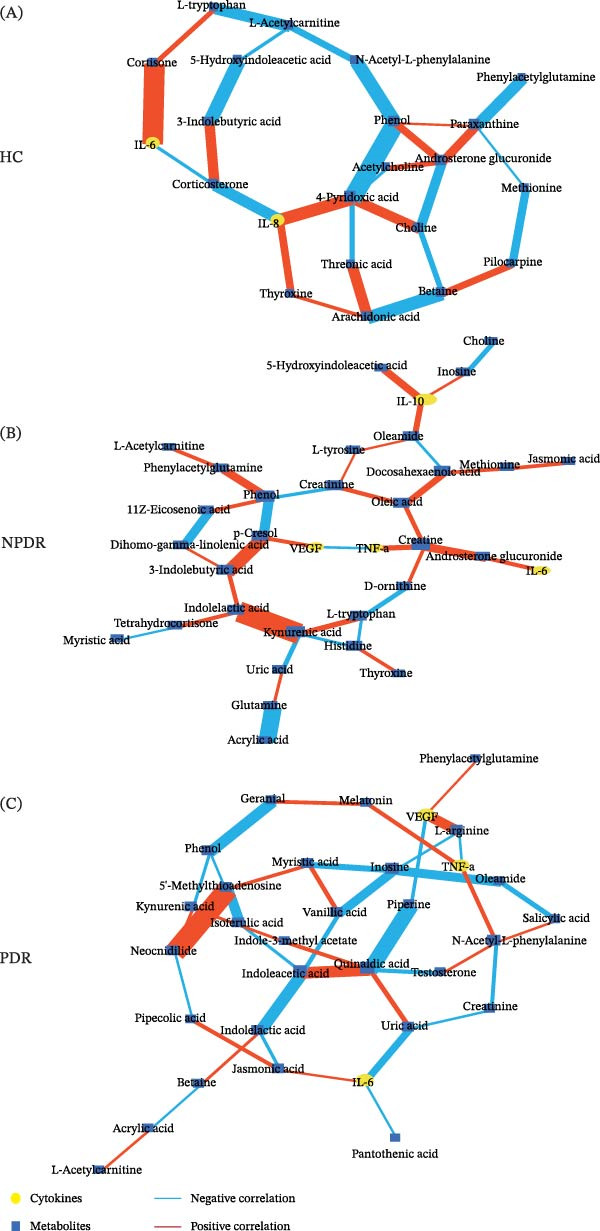
Correlation network between cytokines and metabolites. (A–C) Correlation network diagrams illustrating cytokine–metabolite interactions in (A) HC, (B) NPDR, and (C) PDR groups.

The DSPC network correlation network diagrams (Figure 7A–C) provide a comprehensive visualization of the specific interactions between cytokines and metabolites across each group. In both NPDR and PDR patients, VEGF, TNF‐α, and IL‐6 were critically involved within the cross‐regulatory network, potentially suggesting the association between systemic metabolic perturbations and ocular inflammation.

## 4. Discussion

This study has systematically compared the untargeted metabolomic profiles among normal controls, NPDR, and PDR patients, revealing significant metabolic dysregulation in DR. Our findings demonstrate particularly pronounced metabolic alterations in PDR groups, whereas NPDR showed more subtle changes relative to controls. We identified several dysregulated circulating metabolites in PDR, with indoleacetic acid, methionine, and uric acid being significantly upregulated—remarkably, these metabolites were abundantly present in PDR patients while being nearly undetectable in HC or NPDR subjects. Conversely, creatinine and oleic acid were substantially decreased in PDR patients. Through ROC analysis, we identified reliable biomarkers for distinguishing PDR, including creatinine, glutamine, indoleacetic acid, and methionine. Interestingly, different biomarkers were identified for NPDR detection: oleic acid, myristic acid, palmitoleic acid, kynurenic acid, and indoleacetic acid. Pathway analysis further revealed distinct patterns of upregulated and downregulated metabolic disruptions in NPDR and PDR, respectively. Importantly, we explored the correlation between plasma metabolic dysfunction and ocular inflammation and ischemic status.

DR remains the leading cause of acquired blindness among working‐age populations, constituting a major complication of diabetes. Despite appropriate management of blood glucose and blood pressure to reduce DR risk, some patients still develop retinopathy, emphasizing the need to identify useful biomarkers for risk stratification. Metabolomics, as a discipline studying metabolic responses in organisms, has made significant progress in recent years in elucidating metabolic disturbances and key metabolites in DR [11].

The significantly dysregulated metabolites in PDR, such as methionine, tryptophan, and glutamine, predominantly belong to the amino acid class, which suggests that the alterations in amino acid metabolism are involved in PDR pathogenesis. Previous research has demonstrated that alterations in amino acid metabolism are commonly observed in metabolic and inflammatory diseases [12]. Methionine, an essential amino acid, has been shown to offer metabolic benefits such as reducing obesity, increasing insulin sensitivity, reducing inflammation and oxidative stress, and prolonging lifespan when restricted in diets [13]. The significant increase in methionine in PDR patients may be associated with homocysteine metabolism. One study demonstrated that DR patients exhibit significantly elevated homocysteine levels following methionine loading tests, suggesting that methionine metabolic dysregulation may promote vascular damage through hyperhomocysteinemia [14]. Glutamine plays a crucial role in the retinal microenvironment, where it is synthesized from glutamate by Müller cells and then supplied to neurons for neurotransmitter production [15]. The observed decrease in glutamine levels in PDR patients may reflect a disruption in this balance, potentially leading to glutamate accumulation, reduced glutamine synthetase (GS) activity, and glutamate excitotoxicity. These changes could contribute to oxidative stress, inflammation, and neuronal apoptosis, exacerbating retinal damage in DR [16]. Further exploration of abnormal glutamate metabolism is essential for understanding the pathogenesis of DR.

Uric acid was significantly elevated in PDR patients, consistent with existing literature. A systematic review and meta‐analysis [17] on uric acid and DR demonstrated that DR patients, particularly those with PDR, exhibit significantly higher uric acid levels compared to controls. Elevated uric acid is closely associated with oxidative stress and inflammation, potentially exacerbating retinal damage through promotion of vascular dysfunction and inflammatory processes [18]. Indoleacetic acid was markedly increased in PDR patients. Although specific research on this metabolite in DR is limited, as a metabolite of tryptophan, its elevation may be related to alterations in the gut microbiota [19]. Dysbiosis of gut microbiota has been reported in diabetic patients and may influence DR progression through systemic inflammation [20]. The increase in indoleacetic acid may reflect the systemic impact of metabolic dysregulation and warrants further investigation. The notably reduced proportion of oleic acid in PDR patients aligns with previous literature. A case–control study found that lower oleic acid intake is associated with increased DR risk [21]. Oleic acid possesses anti‐inflammatory properties [22], and its reduction may reflect diminished protective effects of lipid metabolism, increasing the risk of DR progression.

In the present study, integrated metabolomic and cytokine analysis revealed significant correlations between cytokines/VEGF and specific metabolic pathways in PDR patients. The marked elevation of IL‐6 was closely associated with spermidine and spermine biosynthesis and betaine metabolism, suggesting potential roles for these pathways in exacerbating intraocular inflammation. Spermidine and spermine, both polyamines involved in cellular growth and differentiation, have been implicated in DR pathology. Previous research has demonstrated elevated levels of these polyamines in the vitreous of PDR patients [23], potentially serving as markers of proliferative retinal disease. Betaine, known for its anti‐inflammatory properties, mitigates inflammation by inhibiting NF‐κB activity and NLRP3 inflammasome activation [24]. Therefore, the correlation between IL‐6 and these metabolic pathways suggests that dysregulation of polyamine and betaine metabolism may contribute to the inflammatory state observed in PDR.

The increase in IL‐8 correlated with alterations in pantothenate and CoA biosynthesis and beta‐alanine metabolism. Pantothenate, a precursor of coenzyme A, participates in fatty acid metabolism, and its dysregulation may lead to lipid accumulation and inflammation [25, 26]. Beta‐alanine, a precursor of carnosine, possesses antioxidant properties [27]. The correlation between IL‐8 and these pathways indicates that pantothenate and beta‐alanine may influence retinal inflammation through effects on lipid metabolism and antioxidant defense mechanisms.

Decreased IL‐10 was associated with disruptions in steroidogenesis and phosphatidylcholine biosynthesis. Steroidogenesis produces cortisol, which has potent anti‐inflammatory effects [28]. Phosphatidylcholine, a major component of cell membranes, also possesses anti‐inflammatory properties [29]. The reduction in IL‐10 and dysregulation of these pathways may compromise the retina’s ability to counteract inflammation, thereby promoting disease progression.

VEGF was significantly elevated in PDR and correlated with alterations in spermidine and spermine biosynthesis and vitamin B6 metabolism. As previously noted, polyamines are involved in cellular proliferation [23]. Additionally, vitamin B6 deficiency has been associated with vascular complications like diabetic complications [30, 31]. A recent study found that higher vitamin B6 intake correlates with reduced DR risk, further supporting the importance of vitamin B6 metabolism in DR pathology [32]. Metabolic dysregulation of vitamin B6 may promote angiogenesis through effects on homocysteine levels. Interestingly, the association between vitamin B6 metabolism and VEGF suggests that vitamin B6 supplementation might mitigate angiogenesis in DR, providing a potential target for metabolic intervention.

Although our study reveals the important correlation between metabolic dysregulation and inflammatory response in DR, providing a scientific basis for the identification of potential therapeutic targets, we must acknowledge several limitations. First, the relatively small sample size may limit the generalizability of our findings. Insufficient sample size may result in restricted statistical significance of associations between certain variables and potential overfitting in multivariate models, such as PLS‐DA. The samples were exclusively from the People’s Hospital of Guangxi Zhuang Autonomous Region, further limiting the diversity and representativeness of the sample. Secondly, our metabolite coverage is relatively low, possibly due to low database matching rates, reliance on general databases (such as HMDB), and lack of custom libraries, which makes it challenging to identify species‐specific metabolites. Lastly, our study focuses on discovering phenomena and correlations, with the mechanistic details yet to be deeply explored. To further understand the interaction between metabolites and the inflammatory response and their specific impact on retinopathy, subsequent studies need detailed validation through in vivo and in vitro experiments.

## 5. Conclusion

Our study provides a comprehensive metabolomic analysis of DR, revealing distinct metabolic alterations in NPDR and PDR, with PDR exhibiting particularly pronounced metabolic disturbances. ROC analysis identified several metabolic as reliable biomarkers for distinguishing PDR (like creatinine and methionine) and NPDR (like oleic acid and myristic acid), providing a foundation in developing metabolomic‐based risk stratification and diagnostic tools for DR. Integrating metabolomic and cytokine data, we found significant correlations between metabolic dysfunction and ocular inflammation in PDR. Elevated cytokines such as IL‐6, IL‐8, and VEGF were linked to specific metabolism, highlighting the interplay between metabolism and inflammation/ischemia in DR progression.

## Author Contributions

**Diyang Ke**: formal analysis, visualization, writing – original draft, writing – review and editing. **Yu Wu**: formal analysis, visualization, writing ‐ review and editing. **Ling Cui**: funding acquisition, formal analysis, investigation, software, writing – review and editing. **Ningning Tang**: project administration, resources, supervision, writing – review and editing. **Lingjuan Liu**: conceptualization, resources, writing – review and editing. **Daizai Lei**: software, writing – review and editing. **Xiaozhan Zheng**: resources, investigation, software, writing – review and editing. **Li Jiang**: conceptualization, writing – review and editing. **Zhijie Niu**: funding acquisition, software, writing – review and editing. **Shaoyang Zhang**: investigation, writing – review and editing. **Wenjing He**: conceptualization, resources, writing – review and editing. **Chaolan Shen**: conceptualization, resources, writing – review and editing. **Yuanjun Qin**: resources, writing – review and editing. **Haibin Zhong**: conceptualization, resources, supervision, writing – review and editing. **Fan Xu**: conceptualization, resources, supervision, writing – review and editing. **Fen Tang**: conceptualization, funding acquisition, supervision, writing – original draft, writing – review and editing.

## Funding

The work was supported by the Natural Science Foundation of Guangxi Province (Grants 2025GXNSFDA069013 and 2022GXNSFAA035495) and the Major Talent Program of Guangxi Zhuang Autonomous Region.

## Ethics Statement

This study was ethically reviewed and approved by the Ethics Committee of Guangxi Zhuang Autonomous Region People’s Hospital (Number KY‐GZR‐2023‐106).

## Consent

Informed consent to utilize the collected information for research purposes was obtained from all participants.

## Conflicts of Interest

The authors declare no conflicts of interest.

## Supporting Information

Additional supporting information can be found online in the Supporting Information section.

## Supporting information


**Supporting Information 1** File 1: Complete list of DEMs for NPDR and HC groups.

**Supporting Information 2** File 2: Complete list of DEMs for PDR and HC groups.

**Supporting Information 3** File 3: Complete list of DEMs for PDR and NPDR groups.

## Data Availability

The data that support the findings of this study are available from the corresponding author upon reasonable request.
